# Plasma Neurofilament Light for Prediction of Disease Progression in Familial Frontotemporal Lobar Degeneration

**DOI:** 10.1212/WNL.0000000000011848

**Published:** 2021-05-04

**Authors:** Julio C. Rojas, Ping Wang, Adam M. Staffaroni, Carolin Heller, Yann Cobigo, Amy Wolf, Sheng-Yang M. Goh, Peter A. Ljubenkov, Hilary W. Heuer, Jamie C. Fong, Joanne B. Taylor, Eliseo Veras, Linan Song, Andreas Jeromin, David Hanlon, Lili Yu, Arvind Khinikar, Rajeev Sivasankaran, Agnieszka Kieloch, Marie-Anne Valentin, Anna M. Karydas, Laura L. Mitic, Rodney Pearlman, John Kornak, Joel H. Kramer, Bruce L. Miller, Kejal Kantarci, David S. Knopman, Neill Graff-Radford, Leonard Petrucelli, Rosa Rademakers, David J. Irwin, Murray Grossman, Eliana Marisa Ramos, Giovanni Coppola, Mario F. Mendez, Yvette Bordelon, Bradford C. Dickerson, Nupur Ghoshal, Edward D. Huey, Ian R. Mackenzie, Brian S. Appleby, Kimiko Domoto-Reilly, Ging-Yuek R. Hsiung, Arthur W. Toga, Sandra Weintraub, Daniel I. Kaufer, Diana Kerwin, Irene Litvan, Chiadikaobi U. Onyike, Alexander Pantelyat, Erik D. Roberson, Maria C. Tartaglia, Tatiana Foroud, Weiping Chen, Julie Czerkowicz, Danielle L. Graham, John C. van Swieten, Barbara Borroni, Raquel Sanchez-Valle, Fermin Moreno, Robert Laforce, Caroline Graff, Matthis Synofzik, Daniela Galimberti, James B. Rowe, Mario Masellis, Elizabeth Finger, Rik Vandenberghe, Alexandre de Mendonça, Fabrizio Tagliavini, Isabel Santana, Simon Ducharme, Chris R. Butler, Alexander Gerhard, Johannes Levin, Adrian Danek, Markus Otto, Sandro Sorbi, David M. Cash, Rhian S. Convery, Martina Bocchetta, Martha Foiani, Caroline V. Greaves, Georgia Peakman, Lucy Russell, Imogen Swift, Emily Todd, Jonathan D. Rohrer, Bradley F. Boeve, Howard J. Rosen, Adam L. Boxer, on behalf of the ALLFTD and GENFI consortia

**Affiliations:** From the University of California, San Francisco (J.C.R., P.W., A.M.S., Y.C., A.W., S.-Y.M.G., P.A.L., H.W.H., J.C.F., J.B.T., A.M.K., L.L.M., J.K., J.H.K., B.L.M., H.J.S., A.L.B.); UK Dementia Research Centre (C.H., D.M.C., R.S.C., M.B., M.F., C.V.G., G.P., L.R., I.S., E.T., J.D.R.), UCL Institute of Neurology, Queen Square, London; Quanterix Corp (E.V., L.S., A.J., D.H.), Lexington; Novartis Institutes for Biomedical Research Inc (L.Y., A. Khinikar, R.S.), Cambridge, MA; Novartis Pharma AG (A. Kieloch, M.-A.V.), Basel, Switzerland; Bluefield Project to Cure Frontotemporal Dementia (L.L.M., R.P.), San Francisco, CA; Mayo Clinic (K.K., D.S.K., B.F.B.), Rochester, MN; Mayo Clinic (N.G.-R., L.P., R.R.), Jacksonville, FL; University of Pennsylvania (D.J.I., M.G.), Philadelphia; University of California, Los Angeles (E.M.R., G.C., M.F.M., Y.B.); Harvard University/Massachusetts General Hospital (B.D.C.), Boston, MA; Washington University (N.G.), St. Louis, MO; Columbia University (E.D.H.), New York, NY; University of British Columbia (I.R.M., G.-Y.R.H.), Vancouver, Canada; Case Western Reserve University (B.S.A.), Cleveland, OH; University of Washington (K.D.-R.), Seattle; Laboratory of Neuroimaging (A.W.T.), University of Southern California, Los Angeles; Northwestern University (S.W.), Chicago, IL; University of North Carolina (D.I.K.), Chapel Hill; Texas Health Presbyterian Hospital Dallas (D.K.); University of California, San Diego (I.L.); Johns Hopkins Hospital (C.U.O., A.P.), Baltimore, MD; University of Alabama at Birmingham (E.D.R.); University of Toronto (M.C.T., M.M.), Ontario, Canada; Indiana University School of Medicine (T.F.), Indianapolis; Biogen Inc (W.C., J.C., D.L.G.), Cambridge, MA; Erasmus Medical Centre (J.C.v.S.), Rotterdam, the Netherlands; University of Brescia (B.B.), Italy; University of Barcelona (R.S.-V.); Donostia University Hospital (F.M.), San Sebastian, Gipuzkoa, Spain; Clinique Interdisciplinaire de Mémoire (R.L.), Département des Sciences Neurologiques, CHU de Québec; Faculté de Médecine (R.L.), Université Laval, Quebec, Canada; Center for Alzheimer Research (C.G.), Division of Neurogeriatrics, Department of Neurobiology, Care Sciences and Society, Bioclinicum, Karolinska Institutet; Unit for Hereditary Dementias (C.G.), Theme Aging, Karolinska University Hospital, Solna, Sweden; University of Tübingen (M.S.); Center for Neurodegenerative Diseases (DZNE) (M.S.), Tübingen, Germany; Fondazione IRCCS Ospedale Policlinico (D.G.); University of Milan (D.G.), Centro Dino Ferrari, Italy; Department of Clinical Neurosciences and Cambridge University Hospital (J.B.R.), University of Cambridge, UK; University of Western Ontario (E.F.), London, Canada; KU Leuven (R.V.), Belgium; Neurology Service (R.V.), University Hospitals Leuven, Belgium; University of Lisbon (A.d.M.), Portugal; Fondazione IRCCS Istituto Neurologico Carlo Besta (F.T.), Milan, Italy; University of Coimbra (I.S.), Portugal; McGill University (S.D.), Montreal, Québec, Canada; University of Oxford (C.R.B.); Wolfson Molecular Imaging Centre (A.G.), University of Manchester, UK; University of Duisburg-Essen (A.G.), Duisberg; Ludwig-Maximilians-Universität München (J.L., A.D.); German Center for Neurodegenerative Diseases (J.L.), Munich Cluster for Systems Neurology (SyNergy); University of Ulm (M.O.), Germany; and Department of Neuroscience, Psychology, Drug Research and Child Health (S.S.), University of Florence, and IRCCS Fondazione Don Carlo Gnocchi, Florence, Italy.

## Abstract

**Objective:**

We tested the hypothesis that plasma neurofilament light chain (NfL) identifies asymptomatic carriers of familial frontotemporal lobar degeneration (FTLD)–causing mutations at risk of disease progression.

**Methods:**

Baseline plasma NfL concentrations were measured with single-molecule array in original (n = 277) and validation (n = 297) cohorts. *C9orf72*, *GRN*, and *MAPT* mutation carriers and noncarriers from the same families were classified by disease severity (asymptomatic, prodromal, and full phenotype) using the CDR Dementia Staging Instrument plus behavior and language domains from the National Alzheimer's Disease Coordinating Center FTLD module (CDR+NACC-FTLD). Linear mixed-effect models related NfL to clinical variables.

**Results:**

In both cohorts, baseline NfL was higher in asymptomatic mutation carriers who showed phenoconversion or disease progression compared to nonprogressors (original: 11.4 ± 7 pg/mL vs 6.7 ± 5 pg/mL, *p* = 0.002; validation: 14.1 ± 12 pg/mL vs 8.7 ± 6 pg/mL, *p* = 0.035). Plasma NfL discriminated symptomatic from asymptomatic mutation carriers or those with prodromal disease (original cutoff: 13.6 pg/mL, 87.5% sensitivity, 82.7% specificity; validation cutoff: 19.8 pg/mL, 87.4% sensitivity, 84.3% specificity). Higher baseline NfL correlated with worse longitudinal CDR+NACC-FTLD sum of boxes scores, neuropsychological function, and atrophy, regardless of genotype or disease severity, including asymptomatic mutation carriers.

**Conclusions:**

Plasma NfL identifies asymptomatic carriers of FTLD-causing mutations at short-term risk of disease progression and is a potential tool to select participants for prevention clinical trials.

**Trial Registration Information:**

ClinicalTrials.gov Identifier: NCT02372773 and NCT02365922.

**Classification of Evidence:**

This study provides Class I evidence that in carriers of FTLD-causing mutations, elevation of plasma NfL predicts short-term risk of clinical progression.

Blood-based biomarkers are uniquely valuable for therapeutic development because they are easily obtainable and relatively inexpensive.^1^ Frontotemporal lobar degeneration (FTLD) produces behavioral, cognitive, language, and motor deficits that impair the quality of life of patients and caregivers more severely than other forms of dementia.^2^ About 20% to 30% of FTLD cases are familial, and ≈60% of those are caused by autosomal dominant mutations in 3 genes^3^: chromosome 9 open reading frame 72 (*C9orf72*),^4^ progranulin (*GRN*),^5^ and microtubule-associated protein tau (*MAPT*).^6^ Several therapies are poised to begin clinical trials for familial FTLD (f-FTLD) due to these mutations.^7^ Planning such studies is challenging due to the low f-FTLD prevalence and the lack of good clinical endpoints to monitor disease severity and therapeutic response.

Neurofilament light chain (NfL) is a sensitive marker of neurodegeneration.^8^ CSF NfL is elevated in patients with FTLD compared to patients with Alzheimer disease and healthy controls,^9-12^ with concentrations that correlate with disease severity, cognitive function, and disease progression.^13,14^ CSF NfL concentrations normalize on effective treatment in multiple sclerosis^15^ and spinal muscle atrophy,^16^ suggesting that NfL is sensitive to treatment effects. Serum NfL is elevated in FTLD,^17^ and in symptomatic carriers of f-FTLD–causing mutations, concentrations correlate with brain atrophy.^18^ We tested the hypothesis that plasma NfL could identify asymptomatic f-FTLD mutation carriers at high risk of progression to symptomatic disease. We examined baseline plasma NfL differences related to phenotype, genotype, and disease severity and whether it predicts disease progression in 2 independent cohorts.

## Methods

The primary research question was the following: do plasma NfL concentrations identify f-FTLD mutation carriers at risk of clinical progression (Class I level of evidence)?

### Standard Protocol Approvals, Registrations, and Patient Consents

Participants or their caregivers provided written informed consent, and the study procedures were approved by the local Institutional Review Board committees at each of the participating centers. Patients were recruited through the North American multicenter observational studies Longitudinal Evaluation of Familial Frontotemporal Dementia Subjects (LEFFTDS; ClinicalTrials.gov NCT02372773) and Advancing Research and Treatment in Frontotemporal Lobar Degeneration (ARTFL; ClinicalTrials.gov NCT02365922)^19^ and the Genetic Frontotemporal Dementia Initiative (GENFI).^20^

### Participants

Participants were divided into original (LEFFTDS/ARTFL, n = 277) and validation (GENFI, n = 297) cohorts. LEFFTDS/ARTFL is a North American network of 19 clinical research centers. LEFFTDS enrolled members of families with a known mutation in 1 of the 3 major FTLD genes: *C9orf72*, *GRN*, and *MAPT*. ARTFL enrolled participants who met research criteria for an FTLD syndrome and asymptomatic individuals with a family history of an FTLD syndrome, regardless of whether an FTLD-causing mutation had been identified in the family.^19^ On evaluation, some participants with a family history of FTLD were determined to have prodromal disease or mild cognitive or behavioral impairment (MBI/MCI), as defined previously.^21^ GENFI involves 25 research centers across Europe and Canada and enrolls symptomatic carriers of mutations in the 3 major FTLD genes with frontotemporal dementia and those at risk of carrying a mutation because a first-degree relative is a known symptomatic carrier. Both cohorts consisted of participants with available baseline NfL concentrations, known genotype, and CDR Dementia Staging Instrument plus behavior and language domains from the National Alzheimer's Disease Coordinating Center FTLD module (CDR+NACC-FTLD) global and sum of boxes (sb) scores.^21^ Mutation noncarriers with CDR+NACC-FTLD global score >0 were excluded (11 in the original cohort and 22 in the validation cohort). The validation cohort data have been reported previously.^22^ In the original cohort, clinically defined phenotypes included 184 normal (66.7%), 12 mild behavioral impairment (4.3%), 16 mild cognitive impairment (5.8%), 3 amnestic dementia (1.1%), 48 behavioral variant frontotemporal dementia (bvFTD; 17.4%), 7 frontotemporal dementia with amyotrophic lateral sclerosis (FTD/ALS; 2.5%), 4 primary progressive aphasia (PPA; nonfluent or semantic, 1.4%), and 3 corticobasal syndrome (CBS; 1.1%). Participants in the validation cohort included 240 normal (80.8%), 36 bvFTD (12.1%), 6 FTD/ALS (2%), 3 CBS (1%), and 12 PPA (4%). Data on whether there was conversion from asymptomatic to MBI/MCI or full phenotype or from MBI/MCI to full phenotype were available in 221 of 277 participants in the original cohort and in 159 of 297 participants in the validation cohort.

### Clinical Procedures

Participants underwent annual standardized evaluations that included neurologic assessment, caregiver or companion interview, neuropsychological testing, brain MRI, and biofluid collection for up to 3 years in the original cohort and for 2 years in the validation cohort. Clinical scales included CDR+NACC-FTLD global and CDR+NACC-FTLDsb^21^ and Clinical Global Impression of Severity (CGI-S),^23^ which are based on semistructured interviews and provide global measures of clinical severity; Montreal Cognitive Assessment (MoCA); Unified Parkinson's Disease Rating Scale III, Motor Section^24^; Schwab and England Activities of Daily Living (SEADL), for measurement of impairment in activities of daily living^25^; Functional Assessment Scale (FAS), for assessment of impairment in instrumental activities^26^; and Neuropsychiatric Inventory.^27^ CDR+NACC-FTLD and Mini-Mental State Examination (MMSE) were the only severity scales available in the validation cohort. Neuropsychological testing available in both cohorts included the California Verbal Learning Test–Short Form, immediate and delayed recall^28^; the Benson figure recall^29^; forward and backward digit span; number of correct trials; Trail-Making Test Parts A and B (time to completion)^30^; and phonemic and semantic fluency. In the original cohort, blood samples were centrifuged at 1,500*g* at 4°C for 15 minutes. Plasma was aliquoted in 1,000-μl vials and stored at −80°C at the National Centralized Repository for Alzheimer's Disease and Related Dementias. In the validation cohort, blood samples were collected and processed as previously reported.^22^ Genetic screening was conducted to identify FTLD-causing mutations in the *C9orf72*, *GRN*, and *MAPT* genes and *APOE* polymorphisms as described previously.^22,31^

### Plasma NfL Measurement

In the original cohort, plasma NfL concentrations were measured at baseline with single-molecule array technology (Simoa), using the commercially available NF-light digital immunoassay kit (Quanterix, Lexington, MA). Plasma samples were thawed at room temperature (1 cycle), mixed thoroughly, and centrifuged at 14,000*g* for 3 minutes. The supernatant was loaded onto a Quanterix HD-1 Analyzer with a 1:4 specified dilution. Measures were completed in duplicate over a total of 6 batches, each with an 8-point calibration curve tested in triplicate and 2 controls tested in duplicate. Plasma concentrations were interpolated from the calibration curve within the same batch and corrected for the dilution. All samples were quantifiable within the dynamic range of 0.69 to 2,000 pg/mL and with an average coefficient of variation of 6.5%. Measurements were completed using the same platform in 2 centers: Quanterix (n = 226, February 2018) and Novartis Institutes for Biomedical Research (n = 64, July 2018). Samples from a subset of 186 participants were analyzed twice, independently by each center, with plasma NfL concentrations that were highly correlated (*r* = 0.98, *p* < 0.001). The samples analyzed by the 2 centers also had comparable means and SDs (Quanterix 21.8 ± 35 pg/mL and Novartis 20.2 ± 34 pg/mL), and there were no differences in the median plasma NfL concentrations in 2 groups of age-matched asymptomatic noncarrier controls measured separately (Quanterix 6.9 ± 4 pg/mL, n = 38 vs Novartis 6.4 ± 6 pg/mL, n = 50, *p* = 0.6). The center where samples were analyzed was added as a covariate in statistical analyses. Instrument operators were blinded to clinical and genetic information. In the validation cohort, plasma NfL concentrations were measured with the multiplex Simoa Neurology 4-Plex A kit.^22^

### CSF Biomarker Measurements

CSF biomarkers were available in 113 of the 277 participants at baseline in the original cohort only. Using fit-for-purpose immunoassays, CSF samples were analyzed for NfL, tau, phosphorylated tau_181_ (p-tau), neurogranin, and phosphorylated neurofilament heavy chain (p-NfH) at the following dilutions, 1:50, neat, 1:20, neat, and 1:4, respectively. NfL and tau were measured on the Quanterix Simoa HD-1 (catalog Nos. 103186 and 101552, respectively); p-tau was measured with the Innotest kit (catalog No. 81581); neurogranin was measured with the Euroimmun kit (item code EQ-6551-9601-L); and p-NfH was measured on the Protein Simple Ella platform (catalog No. SPCKB-PS-000519). Measurements were conducted by an independent laboratory with operators blinded to clinical data (Biogen, Inc, Cambridge, MA).

### Neuroimaging

Brain MRI was obtained in the original cohort as described previously^32^ within 45 days of plasma collection except for 15 patients for whom images were obtained within >45 days of plasma collection (median 60 days, range 50–423 days). To simplify relationships with plasma NfL and to control for multiple comparisons, bilateral frontal and temporal gray matter lobar composites were created with regions of interest involved in FTLD syndromes. Frontal regions included frontal pole, lateral orbitofrontal cortex, medial orbitofrontal cortex, middle frontal gyrus, pars opercularis, pars orbitalis, pars triangularis, superior frontal gyrus, and precentral gyrus. Anterior cingulate (caudal and rostral) and insula were also included in the frontal composite, given their significant involvement in FTLD.^33^ Temporal regions included banks of the superior temporal sulcus, entorhinal cortex, fusiform gyrus, middle temporal gyrus, parahippocampal cortex, superior temporal gyrus, temporal pole, and transverse temporal gyrus.

### Statistical Analyses

Biofluid measurements, disease status determination, and statistical analyses were performed separately by different investigators. Original and validation cohort data were handled independently. Data were visually explored with boxplots. NfL data were not normally distributed. Group differences in NfL concentrations were determined with nonparametric tests. Log-transformed NfL data were used as outcome in general linear models to determine between-group differences in NfL concentrations corrected for age and sex. Receiver operating characteristic (ROC) curves tested the diagnostic accuracy of plasma NfL concentrations. Combined forward and backward stepwise linear regressions controlling for age, sex, and genotype determined baseline associations between plasma NfL and clinical variables. Starting with minimal models, the stepwise criteria were such that a variable entered a model when *p* < 0.05, and it was removed when *p* ≥ 0.1. For associations with gray matter volumes, total intracranial volume was an additional control variable.^32^ Linear mixed models tested the ability of baseline log plasma NfL to predict change in clinical variables. All models included interaction terms of log plasma NfL with time as a discrete predictor. Models used compound symmetry covariance and random slopes and intercepts and were controlled for by sex, age, genotype, clinical center, and, when modeling prediction of gray matter volumes, total intracranial volume. Models were run with log plasma NfL as a continuous independent variable and subsequently as a categorical independent variable based on cutoff points derived from Youden indices estimated with ROC curves. Models were run separately for each of the disease severity levels defined by the CDR+NACC-FTLD global score: normal or asymptomatic (carriers and noncarriers run independently) (0), MBI/MCI or prodromal disease (0.5), and dementia or full phenotype (≥1).^21^ Model results were corrected for multiple comparisons across dependent variables for a given disease severity level using false discovery rate.^34^ Analyses were done with SPSS Statistics software, version 26 (IBM, Armonk, NY) and GraphPad Prism, version 8.4 (GraphPad, La Jolla, CA).

### Data Availability

Joint ARTFL and LEFFTDS data and biospecimens and GENFI data are available to qualified investigators for replication of the present study results or further projects.

## Results

### Group Differences in Baseline Plasma NfL Concentrations, Original Cohort

**Figure 1:**
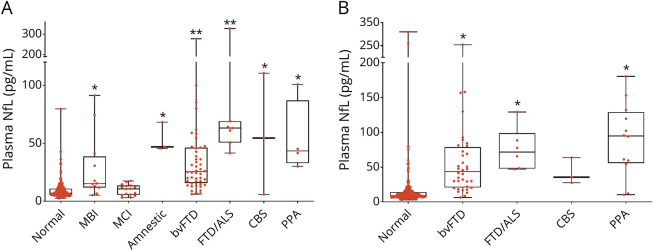
Baseline Plasma NfL Chain Concentrations by Clinical Phenotype (A) Original (Longitudinal Evaluation of Familial Frontotemporal Dementia Subjects [LEFFTDS]/Advancing Research and Treatment in Frontotemporal Lobar Degeneration [ARTFL]) cohort. (B) Validation (Genetic Frontotemporal Dementia Initiative [GENFI]) cohort. Phenotypes are based on clinical diagnosis and did not rely on severity scales. Only the original cohort included clinically diagnosed prodromal disease (mild behavioral impairment [MBI] or mild cognitive impairment [MCI]). Horizontal bars represent median values. Upper and lower quartiles are delimitated by the boxes. Lowest and highest values are indicated by whiskers. bvFTD = behavioral variant frontotemporal dementia; CBS = corticobasal syndrome; FTD/ALS = frontotemporal dementia with amyotrophic lateral sclerosis; NfL = neurofilament light chain; PPA = primary progressive aphasia (nonfluent or semantic). *Compared to normal. **Compared to normal and MCI, *p* < 0.05.

Of 277 individuals with baseline evaluations (table 1), 221 (79.7%) and 148 (53.4%) also had follow-up data available for years 1 and 2, respectively. In all genotypes combined and after correction for age and sex, amnestic dementia, bvFTD, FTD/ALS, CBS, and PPA phenotypes had higher plasma NfL concentrations than asymptomatic participants (mutation carriers and noncarriers combined) and those with MCI (figure 1).

**Table 1:**
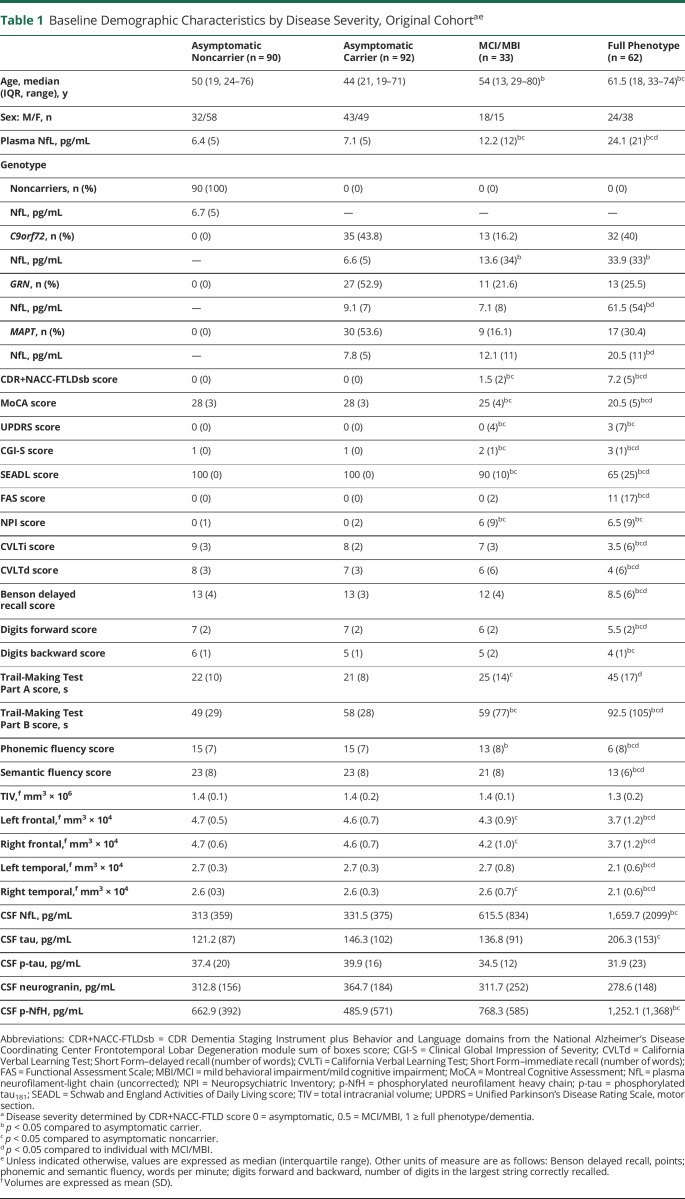
Baseline Demographic Characteristics by Disease Severity, Original Cohort^ae^

**Figure 2:**
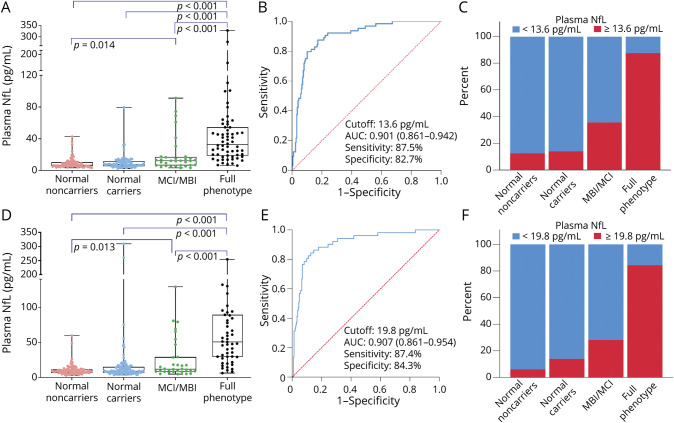
Baseline Plasma NfL Concentrations by Disease Severity and Diagnostic Performance (A–C) Original (Longitudinal Evaluation of Familial Frontotemporal Dementia Subjects [LEFFTDS]/Advancing Research and Treatment in Frontotemporal Lobar Degeneration [ARTFL]) cohort. (D–F) Validation (Genetic Frontotemporal Dementia Initiative [GENFI]) cohort. Severity was determined by the CDR Dementia Staging Instrument plus Behavior and Language domains from the National Alzheimer's Disease Coordinating Center Frontotemporal Lobar Degeneration module (CDR+NACC-FTLD). (A and D) Boxplots show plasma neurofilament light chain (NfL) concentrations in asymptomatic carriers (i.e., CDR+NACC-FTLD score 0), those with mild behavioral or cognitive impairment (mild behavioral impairment/mild cognitive impairment [MBI/MCI], CDR+NACC-FTLD score 0.5), and patients with full phenotypes (CDR+NACC-FTLD score ≥1). Horizontal bars represent median values. Upper and lower quartiles are delimitated by the boxes. Lowest and highest values are indicated by whiskers. (B and E) Receiver operating characteristic (ROC) curves show that plasma NfL was a good discriminator between individuals with full phenotype and those either asymptomatic or with MBI/MCI. (C and F) Proportion of patients with low or high plasma NfL concentrations, determined by the ROC curve, is presented for each disease severity. AUC = area under the curve.

**Figure 3:**
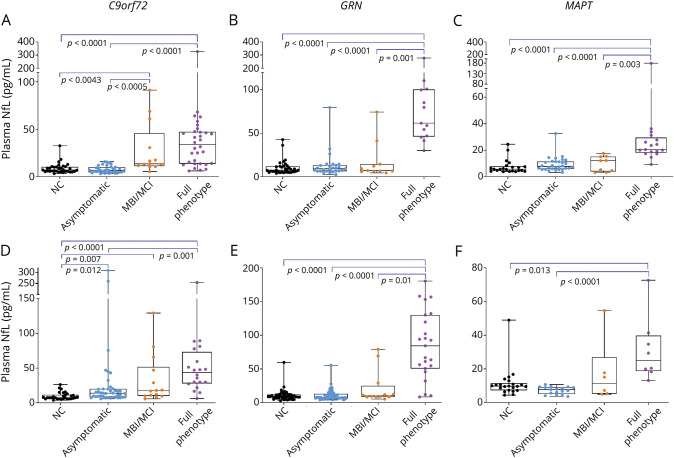
Plasma NfL Concentrations by Disease Severity in Each Genotype Group (A–C) Original cohort. (D–F) Validation cohort. MBI/MCI = mild behavioral or cognitive impairment (CDR Dementia Staging Instrument plus Behavior and Language domains from the National Alzheimer's Disease Coordinating Center Frontotemporal Lobar Degeneration module score 0.5); *C90rf72* = chromosome 9 open reading frame 72; *GRN* = progranulin; *MAPT* = microtubule-associated protein tau; NC = noncarrier; NfL = neurofilament light chain.

As defined by disease severity, 65.7% of the participants (33.2% carriers and 32.5% noncarriers) were asymptomatic (CDR+NACC-FTLD score 0), 11.9% had MBI/MCI (CDR+NACC-FTLD score 0.5), and 22.4% had full phenotype (CDR+NACC-FTLD score ≥1). Median baseline plasma NfL concentrations were highest in participants with full phenotype (figure 2). There were no differences in NfL concentrations between asymptomatic mutation carriers and noncarriers for any genotype. Median plasma NfL concentrations tended to be higher in those with MBI/MCI than asymptomatic mutation carriers, but the results did not reach statistical significance (12.2 ± 10 pg/mL vs 7.5 ± 6 pg/mL, *p* = 0.085, mean estimate difference 0.44, 95% confidence interval [CI] 0.85–0.99, *p* = 0.016) in all genotypes combined. In *C9orf72* carriers, NfL concentrations were higher in participants with MBI/MCI compared to asymptomatic individuals (13.6 ± 34 pg/mL vs 6.6 ± 5 pg/mL, *p* < 0.001, figure 3) but not in *GRN* or *MAPT*. There were no genotype-related differences in NfL in asymptomatic mutation carriers or those with MBI/MCI. In full phenotype, NfL was higher in *GRN* (61.5 ± 54 pg/mL) than in *C9orf72* (33.9 ± 33 pg/mL, *p* < 0.001) and *MAPT* (20.5 ± 11 pg/mL, *p* < 0.001).

In all participants combined, a cut point of ≥13.6 pg/mL discriminated individuals with full phenotype from asymptomatic individuals or those with MBI/MCI with 87.5% sensitivity, 82.7% specificity, 59.7% positive predictive value, and 96.2% negative predictive value (area under the curve [AUC] 0.901, 95% CI 0.861–0.942, *p* < 0.001). Plasma NfL was a poor discriminator between asymptomatic mutation carriers and those with MBI/MCI (AUC 0.676, 95% CI 0.588–0.724, *p* < 0.001), but it was a better discriminator between participants with MBI/MCI and those with full phenotype (0.803, 95% CI 0.744–0.862, *p* < 0.001). The proportion of participants with high (≥13.6 pg/mL) NfL differed by severity group: 12.2% in asymptomatic mutation noncarriers, 14.1% in asymptomatic mutation carriers, 39.4% in those with MBI/MCI, and 88.7% in those with full phenotype (χ^2^ = 119.6, *p* < 0.001).

### Baseline Correlations With Clinical Variables, Original Cohort

Baseline NfL strongly correlated with age in the overall sample (ρ = 0.69, 95% CI 0.505–0.695, *p* < 0.001) and in asymptomatic individuals (ρ = 0.63, 95% CI 0.437–0.769, *p* < 0.001) and those with MBI/MCI (ρ = 0.71, 95% CI 0.364–0.917, *p* < 0.001); it correlated weakly in individuals with full phenotype (ρ = 0.23, 95% CI −0.109 to 0.402, *p* = 0.07). NfL concentrations were higher in women than in men (10.7 ± 13 pg/mL vs 7.6 ± 9 pg/mL, mean estimate difference 0.75, 95% CI 0.59–0.95, *p* = 0.01), even after controlling for age, disease severity, and genotype (β = 0.251, 95% CI 0.092–0.409, *p* = 0.002). In all participants, plasma NfL was strongly associated with all clinical, neuropsychological, and gray matter volume variables at baseline. None of the relationships were affected by genotype, and they remained essentially unchanged after exclusion of asymptomatic noncarriers (eTable 1, doi.org/10.7272/Q6W957CZ). The strongest associations were observed with measures of disease severity, including CDR+NACC-FTLDsb, CGI-S, SEADL, and FAS scores. Weaker associations were observed with gray matter volumes. CSF biomarkers were available in 113 (40.7%) participants (34 asymptomatic noncarriers, 46 asymptomatic mutation carriers, 14 with MBI/MCI, and 19 with full phenotype). Plasma NfL correlated with CSF NfL (ρ = 0.74, *p* < 0.001), CSF p-NfH (ρ = 0.73, *p* < 0.001), and CSF tau (ρ = 0.45, *p* < 0.001), but not with CSF neurogranin (ρ = 0.06, *p* = 0.94) or CSF p-tau (ρ = 0.07, p = 0.46). There were no differences in the proportion of *APOE* carriers as a function of clinical phenotype, genotype, or disease severity or differences in NfL concentrations by *APOE* genotype.

### Baseline NfL, Phenoconversion, and Disease Progression, Original Cohort

**Figure 4:**
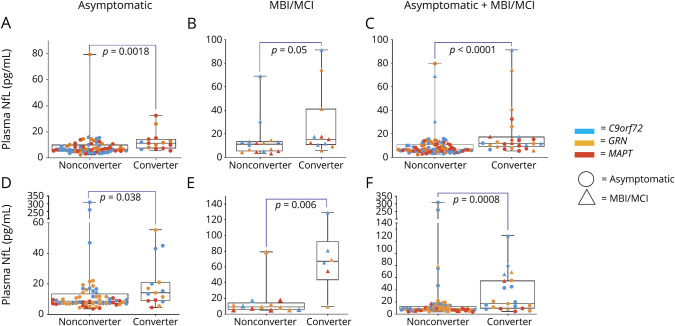
Baseline Plasma NfL Concentrations According to Conversion Status by Follow-Up Severity was determined with the CDR Dementia Staging Instrument plus Behavior and Language domains from the National Alzheimer's Disease Coordinating Center Frontotemporal Lobar Degeneration module (CDR+NACC-FTLD). (A–C) Original (Longitudinal Evaluation of Familial Frontotemporal Dementia Subjects [LEFFTDS]/Advancing Research and Treatment in Frontotemporal Lobar Degeneration [ARTFL]) cohort. (D–F) Validation (Genetic Frontotemporal Dementia Initiative [GENFI]) cohort. (A and D) Median baseline neurofilament light chain (NfL) concentrations were higher in asymptomatic mutation carriers (CDR+NACC-FTLD score 0) who progressed to either mild behavioral or cognitive impairment (MBI/MCI; CDR+NACC-FTLD score 0.5) or full phenotype (CDR+NACC-FTLD score ≥1) on follow-up. (B and E) A similar trend was observed in individuals who had MBI/MCI at baseline and when all participants (asymptomatic mutation carriers and those with MBI/MCI) were combined (C and F). Horizontal bars represent median values. Upper and lower quartiles are delimitated by the boxes. Lowest and highest values are indicated by whiskers. Circles = asymptomatic; triangles = MBI/MCI; blue = chromosome 9 open reading frame 72(*C9orf72*) mutation carriers; red = microtubule-associated protein tau (*MAPT*) mutation carriers; yellow = progranulin (*GRN*) mutation carriers;.

Twenty-six mutation carriers phenoconverted after 2 years (15 asymptomatic [12 to MBI/MCI and 3 to full phenotype] and 11 MBI/MCI to full phenotype). Phenoconversion occurred in 10 of 21 (47.6%) of asymptomatic or MBI/MCI mutation carriers with baseline NfL ≥13.6 pg/mL compared to 16 of 84 (11.4%) of those with baseline NfL <13.6 pg/mL (*p* = 0.007). Median baseline NfL concentrations were higher in asymptomatic mutation carriers who phenoconverted to either MBI/MCI or dementia over the next 2 years compared to those who remained asymptomatic (11.4 ± 7 pg/mL vs 6.7 ± 5 pg/mL, *p* = 0.002, figure 4). Plasma NfL concentrations were also higher in asymptomatic mutation carriers whose CDR+NACC-FTLDsb scores progressed by 1 point, even in the absence of phenoconversion (10.8 ± 8 pg/mL), compared to those whose scores remained stable (6.6 ± 3 pg/mL, *p* = 0.0017, data available from Dryad, efigure 1, doi.org/10.7272/Q6W957CZ).

#### Asymptomatic Mutation Carriers

**Figure 5:**
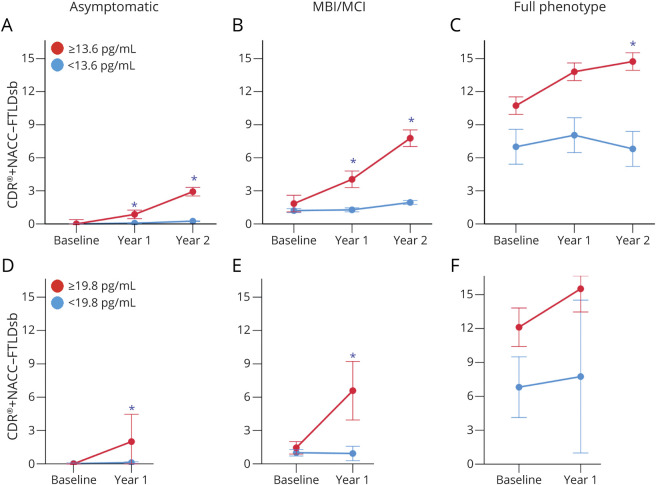
Prediction of Clinical Progression by Plasma NfL in Familial Frontotemporal Lobar Degeneration (A–C) Original (Longitudinal Evaluation of Familial Frontotemporal Dementia Subjects [LEFFTDS]/Advancing Research and Treatment in Frontotemporal Lobar Degeneration [ARTFL) cohort. (D–F) Validation (Genetic Frontotemporal Dementia Initiative [GENFI]) cohort. Figure shows the results of models using data from all genotypes in each severity group. In the original cohort, patients with high (red; ≥13.6 pg/mL) baseline plasma neurofilament light chain (NfL) showed worse clinical scores at 2 years compared to patients with low (blue; <13.6 pg/mL) NfL, which was supported by NfL level–by–time interaction. This differential predictive effect by NfL level was observed regardless of disease severity, including asymptomatic carriers. Similar results were observed in the validation cohort with a cut point value of 19.8 pg/mL. CDR+NACC-FTLDsb = CDR Dementia Staging Instrument plus Behavior and Language domains from the National Alzheimer's Disease Coordinating Center Frontotemporal Lobar Degeneration module sum of boxes score. *Between-group contrast at that time point, *p* < 0.05.

As a continuous variable, baseline NfL related to future decline in CDR+NACC-FTLDsb, CGI-S, and FAS scores (table 2). For example, every baseline log NfL 1 pg/mL in asymptomatic mutation carriers was associated with a 1.6-point increase in CDR+NACC-FTLDsb score at year 1 (95% CI 0.75–2.6, *p* < 0.001) and a 2.5-point increase at year 2 (95% CI 1.6–3.4, *p* < 0.001). Similar results were observed when NfL was analyzed as a categorical variable. For example, asymptomatic mutation carriers with high (≥13.6 pg/mL) baseline NfL had CDR+NACC-FTLDsb scores were 1.6 points higher at 1 year (95% CI 1.0–2.2, *p* < 0.001) and 2.4 points higher at 2 years (95% CI 1.8–3.0, *p* < 0.001) than those with low baseline NfL (figure 5). High NfL also related to lower frontal and temporal brain volumes after 2 years. NfL did not predict change in any of the clinical scales or brain volumes in mutation noncarriers.

**Table 2:**
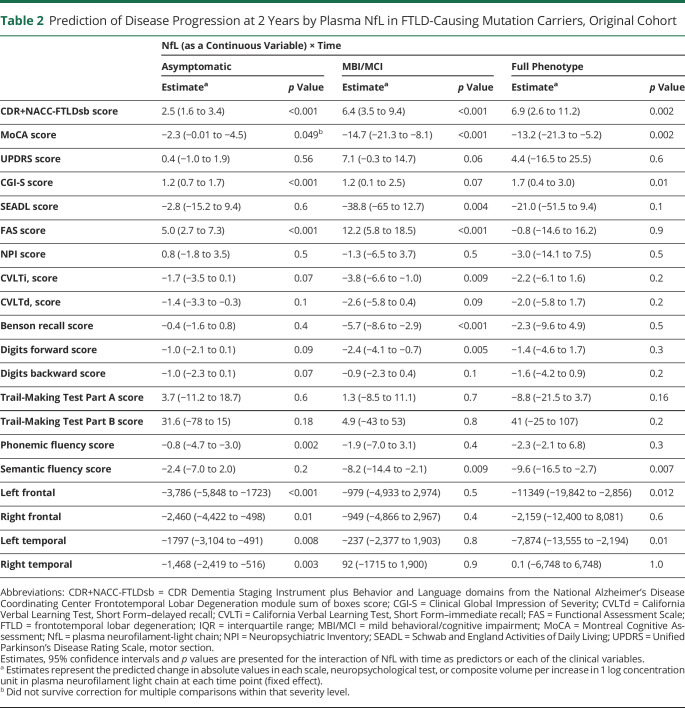
Prediction of Disease Progression at 2 Years by Plasma NfL in FTLD-Causing Mutation Carriers, Original Cohort

#### Individuals With MBI/MCI

In mutation carriers with MBI/MCI at baseline (CDR+NACC-FTLD score 0.5), baseline NfL was strongly associated with decline at year 2 on CDR+NACC-FTLDsb, MoCA, SEADL, FAS, California Verbal Learning Test immediate recall, Benson recall, digits forward, and semantic fluency scores, but not in brain volumes (table 2).

#### Full Phenotype

In mutation carriers with full phenotype (CDR+NACC-FTLD score ≥1), baseline NfL related to decline in CDR+NACC-FTLDsb, MoCA, and SEADL phonemic fluency scores and brain volume composites after 2 years (table 2).

### Validation Cohort

In the validation cohort, of 297 participants with baseline evaluations, 189 (63.6%) had follow-up year 1 data (available in Dryad, eTable 2, doi.org/10.7272/Q6W957CZ). Plasma NfL concentrations were higher in all symptomatic mutation carriers compared to asymptomatic participants except for CBS (figure 1). Median baseline plasma NfL concentrations were higher in participants with full phenotype (50.6 ± 59 pg/mL) compared to asymptomatic mutation noncarriers (8.8 ± 5 pg/mL), asymptomatic mutation carriers (9.1 ± 8 pg/mL), and those with MBI/MCI (12.1 ± 20 pg/mL, *p* < 0.001) (figure 2). A cut point of ≥19.8 pg/mL discriminated those with full phenotype from asymptomatic individuals or those with MBI/MCI with 87.4% sensitivity, 84.3% specificity, 58.1% positive predictive value, and 96.4% negative predictive value (AUC 0.907, 95% CI 0.861–0.954, *p* < 0.001). This cut point was also a fair discriminator between MBI/MCI and full phenotype (AUC 0.805, 95% CI 0.704–0.906) but not between asymptomatic mutation carriers and those with MBI/MCI (AUC 0.641, 95% CI 0.530–0.752). The proportion of participants with high (≥19.8 pg/mL) NfL was different in each disease severity group (6.1% in asymptomatic mutation noncarriers, 13.9% in asymptomatic mutation carriers, 28.1% in those with MBI/MCI, and 84.3% in individuals with full phenotype, χ^2^ = 122.6, *p* < 0.001). In the whole cohort or in mutation carriers only, baseline plasma NfL correlated with CDR+NACC-FTLDsb score, MMSE score, and all neuropsychological measures (eTable 2, doi.org/10.7272/Q6W957CZ).

Twenty-one mutation carriers phenoconverted after 1 year (15 asymptomatic individuals [13 to MBI/MCI and 2 to full phenotype] and 6 with MBI/MCI to full phenotype). Plasma NfL concentrations were higher in phenoconverters than nonphenoconverters in asymptomatic mutation carriers (14.1 ± 12 pg/mL vs 8.7 ± 6 pg/mL, *p* = 0.038) and those with MBI/MCI (67.3 ± 49 pg/mL vs 9.0 ± 8 pg/mL, *p* = 0.006) (figure 4). Plasma NfL concentrations were also higher in asymptomatic mutation carriers whose CDR+NACC-FTLDsb scores progressed by 1 point, even in the absence of phenoconversion (15.3 ± 33 pg/mL) compared to those whose scores remained stable (8.9 ± 7 pg/mL, *p* = 0.014, efigure 1, doi.org/10.7272/Q6W957CZ). In asymptomatic mutation carriers, baseline NfL predicted worsening at year 1 in CDR+NACC-FTLDsb, MMSE, and Trail-Making Test Part A scores. In participants with MBI/MCI, baseline NfL predicted decline at year 1 in CDR+NACC-FTLDsb, MMSE, Trail-Making Test Part B, and phonemic fluency scores. In those with full phenotype, baseline NfL was associated with subsequent decline in MMSE and Trail-Making Test Part A scores, but the relationships did not survive correction for multiple comparisons (available in Dryad, eTable 3).

## Discussion

We analyzed the prognostic value of plasma NfL concentrations in carriers of the most common FTLD-causing mutations, *C9orf72*, *GRN*, and *MAPT,* over 1–2 years of follow-up, with a special emphasis on asymptomatic mutation carriers and carriers with prodromal disease (MBI/MCI). In 2 independent cohorts, plasma NfL concentrations were strongly related to disease severity with stepwise increases from asymptomatic (clinically normal) through MBI/MCI to full phenotype. At baseline, plasma NfL was strongly correlated with global and functional status, neuropsychological scores, and brain volume. Higher baseline NfL was associated with greater disease severity after 1 or 2 years of follow-up, regardless of disease severity and genotype. Remarkably, this included asymptomatic mutation carriers, in whom plasma NfL was also associated with future clinical decline, allowing identification of individuals at high risk for phenoconversion to symptomatic status within 2 years. Consistent with this finding, NfL also predicted worse clinical and neuropsychological status or more brain atrophy, regardless of disease severity and genotype. These results suggest a role for plasma NfL as a prognostic biomarker in f-FTLD.

The findings in our original and validation cohorts are consistent with previous studies of serum NfL in f-FTLD and sporadic FTLD. In f-FTLD, serum NfL is associated with disease severity, brain volume, and brain atrophy.^18^ In symptomatic sporadic FTLD, baseline serum NfL correlated with executive function and brain atrophy, but not with longitudinal change in neuropsychological scores,^17^ which is similar to what we observed in participants with full phenotype. This study and others^18,22,35^ found that in fully symptomatic patients, *GRN* mutation carriers had higher NfL concentrations than *C9or72* and *MAPT* mutation carriers. This does not seem to be due to differences in the number of participants by genotype or the age of symptomatic participants in each genetic group and may reflect a faster rate of neurodegeneration in symptomatic *GRN* mutation carriers. Consistent with previous studies, we observed baseline NfL differences between symptomatic and asymptomatic FTLD mutation carriers and between phenoconverters and nonconverters.^35^ Similar to those studies, we also observed a large within-group variability in NfL concentrations, regardless of clinical phenotype, disease severity, or genotype. This variability likely explains why median NfL concentrations in asymptomatic mutation carriers were not elevated, yet high concentrations were still associated with future clinical progression. In this group, NfL showed good negative predictive value but poor positive predictive value for phenoconversion. The absolute cutoff values for discrimination between asymptomatic and symptomatic participants were similar to those reported in previous studies based on data from our validation cohort.^17,18,35^ However, 1 study reported a higher cutoff (33 pg/mL)^17^ that may be explained by the inclusion of older controls and sporadic cases compared to the familial cases reported here.^36^

Unlike previous studies, we used the CDR+NACC-FTLD score to stratify patients by level of global impairment, allowing delineation of MBI/MCI, a prodromal state of mild or questionable disease between asymptomatic and full phenotype. The CDR+NACC-FTLD score is more appropriate for patients with FTLD and superior to relying on the clinical phenotype or the traditional Clinical Dementia Rating because the CDR+NACC-FTLD includes measures of behavioral and language impairment.^37^ We found that baseline NfL concentrations in asymptomatic and MBI/MCI mutation carriers best predicted changes in global and functional scales (i.e., CDR+NACC-FTLDsb, CGI-S, and FAS). In addition, NfL predicted declines in activities of daily living, as measured by the SEADL and FAS scales and several neuropsychological tests, in individuals with MBI/MCI, but not in asymptomatic mutation carriers or full phenotype. The severity-dependent differences in predictive value of baseline NfL are probably attributable to a number of factors. These include a faster rate of functional decline in MBI/MCI, differences in the duration of the MBI/MCI stage depending on the phenotype, and absence of activities of daily living impairments in asymptomatic individuals and a ceiling effect for deterioration in fully symptomatic individuals. Identification of individuals with MBI/MCI, however, may be challenging. The sample sizes for MBI/MCI in both cohorts of this study were relatively small, and the follow-up durations were limited. This may explain why differences in baseline NfL concentrations in participants with MBI/MCI by conversion status were not as strong compared to differences between those with MBI/MCI and asymptomatic or fully symptomatic mutation carriers. These observations might also reflect a short duration in the MBI/MCI state and fluctuation in clinical status over time, with some participants with MBI/MCI progressing to full phenotype and others returning to asymptomatic status. The additional follow-up data that will be collected as part of the ongoing ARTFL LEFFTDS Longitudinal Frontotemporal Dementia (ALLFTD) study^38^ will improve the understanding of the clinical value of plasma NfL in prodromal f-FTLD.

Our results suggest that plasma NfL may be a promising endpoint for FTLD clinical trials. A variety of therapies that target the underlying pathologic proteins encoded by the 3 FTLD-causing genes studied here are entering clinical trials for f-FTLD.^7^ The ultimate goal for these therapies is to prevent disease onset in mutation carriers. A major challenge for testing the efficacy of such interventions is the inability to measure clinically meaningful endpoints in asymptomatic individuals who are at risk for disease. Recent US Food and Drug Administration guidance on developing therapeutics for presymptomatic or early Alzheimer disease suggests that therapies might be approved under an accelerated mechanism on the basis of a biomarker that is “reasonably likely to predict clinical benefit.”^39^ Our data show associations between plasma NfL concentrations and subsequent functional status, which are considered inherently clinically meaningful, within 2 years of follow-up. Therefore, plasma NfL might be used as a continuous variable endpoint (difference in mean NfL concentration in placebo vs intervention arm) or as a time-to-event endpoint (delay in onset of the sharp rise in NfL that occurs at the transition from the asymptomatic to symptomatic phase of disease). Such an approach was previously used for drugs to treat macular degeneration that were approved for marketing by using optical coherence tomography measurements as endpoints that are highly predictive of future declines in visual acuity.^40^

Our study has limitations. NfL is not a pathophysiology-specific biomarker of FTLD, and its elevations in a number of general conditions render it a nonspecific marker of neuronal injury. Future projects should aim at identifying and deploying specific markers of disease activity and severity in FTLD, and we have previously reported the comparative diagnostic value of plasma NfL vs plasma p-tau in FTLD and Alzheimer disease.^41^ On the basis of work in dominantly inherited Alzheimer disease,^42^ longitudinal plasma NfL measurements may have better predictive ability for clinical decline than the cross-sectional measures we used. Longitudinal plasma samples of participants of the LEFFTDS and ARTFL projects are being collected, and future projects will examine longitudinal NfL concentrations and their relationship with disease progression. Finally, we found no influence of the *APOE* genotype on NfL concentrations or predictive ability. The analyses, however, did not examine other potential genetic risk factors such as polymorphisms within *MAPT*,^43^ *TMEM106B*,^44^ or *EGFR*^45^ that have been identified as potential modulators of FTLD risk.

This study adds to a large body of evidence supporting plasma NfL as a useful prognostic biomarker for syndromes associated with FTLD.^12,14,17,35,46,47^ By demonstrating the ability to identify asymptomatic FTLD mutation carriers at risk of progression to symptomatic status over 2 years, our findings provide a strong rationale for developing this biomarker as a potential inclusion criterion or endpoint for prevention studies in asymptomatic f-FTLD mutation carriers.

## Glossary

ALLFTD: ARTFL LEFFTDS Longitudinal Frontotemporal Dementia
ARTFL: Advancing Research and Treatment in Frontotemporal Lobar Degeneration
AUC: area under the curve
bvFTD: behavioral variant frontotemporal dementia
CBS: corticobasal syndrome
CDR+NACC-FTLD: CDR Dementia Staging Instrument plus Behavior and Language domains from the National Alzheimer’s Disease Coordinating Center Frontotemporal Lobar Degeneration module
CGI-S: Clinical Global Impression of Severity
*C90rf72*: chromosome 9 open reading frame 72
CI: confidence interval
FAS: Functional Assessment Scale
FTLD: frontotemporal lobar degeneration
f-FTLD: familial FTLD
FTD/ALS: frontotemporal dementia with amyotrophic lateral sclerosis
GENFI: Genetic Frontotemporal Dementia Initiative
*GRN*: progranulin
LEFFTDS: Longitudinal Evaluation of Familial Frontotemporal Dementia Subjects
*MAPT*: microtubule-associated protein tau
MBI/MCI: mild behavioral or cognitive impairment
MMSE: Mini-Mental State Examination
MoCA: Montreal Cognitive Assessment
NfL: neurofilament light chain
p-NfH: phosphorylated neurofilament heavy chain
PPA: primary progressive aphasia
p-tau: phosphorylated tau181
ROC: receiver operating characteristic
sb: sum of boxes
SEADL: Schwab and England Activities of Daily Living
Simoa: single-molecule array
